# Technological evaluations: the need for evidence-informed healthcare

**DOI:** 10.1590/S1516-31802007000100001

**Published:** 2007-01-04

Considering that health sector costs have historically gone up by amounts ranging from 15% to 20% per year and that countries have had increases in gross domestic product (GDP) of only around 5% (and some have had much less), it is not necessary to be a great mathematician or guru to foresee that, in less than 20 years from now, their health systems will be bankrupt. The present expenditure on health (around 1.7 trillion dollars per year in the United States) is already insufficient. The 10 or 20 billion dollars spent in Brazil will increasingly mean that, as the writer Millôr Fernandes would put it, there will be many months left after the country's last dollar. This scenario will, unless the capacity to seek efficiency in the health sector is boosted worldwide, lead to a situation in which the private sector will tend to change its activities and the public sector will do what is possible with the insufficient resources that remain for it. In other words, in terms of investments in health, what the United States is today, Brazil will tend to be tomorrow. And with another disadvantage: its health professionals will no longer be accustomed to offering medical care with the same altruism as here today. And there, the greed of judicial demands will continue unchanged, causing doctors to be more concerned about not facing lawsuits and penalties than for caring for patients, and therefore to practice what is called defensive medicine.

What can be done? On the one hand, quality evidence-based preventive medicine can be developed. Emphasis can be given to education and sanitation measures, and so on… On the other hand, health professionals can be given critical capacity and continuing access to countries’ state-of-the-art information, and their health systems can have the capacity and competence to utilize technological evaluations in the field of health.

Medical schools should create disciplines of clinical epidemiology, evidence-based medicine and health economics, so that new generations of doctors and health professionals can graduate already understanding the basic concepts for seeking effectiveness, efficiency and safety. Knowing at least how to use the Cochrane Library would be a good start.^1^

There is no doubt today that every health department, hospital and health insurance company would now be greatly benefited through the assistance of a professional with skills in evidence-based medicine. New data that have not yet been published show that around 20% of medical schools are already officially teaching evidence-based medicine in their curricula. Although these data are promising, it can be supposed that the other 80% may be swimming against the tide of history and training professionals without the critical skills to make technological evaluations, who thus become easy victims for laboratory representatives, commercial interests, etc.

The professional bodies representing the medical profession in Brazil — Associação Paulista de Medicina (APM), Associação Médica Brasileira (AMB) and Conselho Federal de Medicina (CFM), some health insurers, like the UNIMED chain, the Ministry of Health (Department of Science and Technology, DECIT), the National Agency for Supplementary Health (ANS), the National Agency for Sanitary Surveillance (ANVISA), the World Health Organization (WHO) and members of the Federal Public Attorneys’ Office have already recognized the relevance of this matter in facing up to the challenges. Universities cannot just be pulled along in this process. Acquisition of critical appraisal skills for conducting technological evaluations in health has a fundamental strategic role in the application of science, in which the basic objective should always be the search for health and improvement of the quality of life for humanity and environmental preservation. It is necessary to understand, like Brazilian Indians do, that life depends on the preservation of nature: they consider life, in all its manifestations, as sacred, and that to respect life is to respect the most sacred things there are.
